# Blind Estimation Methods for BPSK Signal Based on Duffing Oscillator

**DOI:** 10.3390/s20226412

**Published:** 2020-11-10

**Authors:** Ke Wang, Xiaopeng Yan, Zhiqiang Zhu, Xinhong Hao, Ping Li, Qian Yang

**Affiliations:** 1 Science and Technology on Electromechanical Dynamic Control Laboratory, School of Mechatronical Engineering, Beijing Institute of Technology, Beijing 100081, China; 3120170161@bit.edu.cn (K.W.); haoxinhong@bit.edu.cn (X.H.); liping85@bit.edu.cn (P.L.); yangqian@bit.edu.cn (Q.Y.); 2 Beijing Institute of Electronic System Engineering, Beijing 100854, China; xxxxmtb@163.com

**Keywords:** Duffing oscillator, BPSK signal, blind estimation, low SNRs

## Abstract

To realize the blind estimation of binary phase shift keying (BPSK) signal, this paper describe a new relational expression among the state of Duffing oscillator excited by BPSK signal, the pseudo-random code of BPSK signal, and the difference frequency between the to-be-detect signal and internal drive force signal of Duffing oscillator. Two output characteristics of Duffing oscillators excited by BPSK signals named implied periodicity and pilot frequency array synchronization are presented according to the different chaotic states of Duffing oscillator. Then two blind estimation methods for the carrier frequency and pseudo-random sequence of the BPSK signal are proposed based on these two characteristics, respectively. These methods are shown to have a significant effect on the parameter estimation of BPSK signals with no prior knowledge, even at very low signal-to-noise ratios (SNRs).

## 1. Introduction

In modern radar electronic warfare, reconnaissance and anti-reconnaissance efforts, as well as the evaluations of the reconnaissance effect, are of great importance. Obtaining useful information from enemy radar signals can provide guidance for radar jamming and fire destruction and enable effective countermeasures to be taken against enemy interference [1,2]. In the field of radar and communication, binary phase shift keying (BPSK) signals are commonly used because of their high frequency band utilization, strong anti-noise interference ability, wide signal bandwidth, and resistance to detection [3,4,5,6]. In actual battlefield situations, there is often a complex electromagnetic environment and electronic countermeasures under non-cooperative conditions, and so useful signals are often drowned in strong noise, complicating the tasks of efficient intercepted efficiently and accurate estimated. For these reasons, blind detection of the weak BPSK signals at low signal-to-noise ratios (SNRs) is an important subject.

Part of the literatures focuses on the estimation of carrier frequency. Methods based on the cyclic spectrum density [7], second-order cyclic statistics [8], and stochastic resonance (SR) [9] have been developed to estimate carrier frequency and symbol rate of BPSK signal, but these methods cannot calculate the pseudo-random code, and their estimation accuracy is poor in low SNR environments (the SNR should be greater than −6 dB, −2 dB, and 0 dB, respectively). The other part of the research estimates the pseudo-random sequence. Wang et al. [10] estimate the period and staring bit of pseudo-random sequence through the method of reprocessing the power spectrum density, segmentation processing, and an average cross-correlation calculation, which can be realized even the SNR = −15 dB, but the carrier frequency needs to be known in advance. Synchronous demodulation method can identify pseudo-random codes effectively and provide easier parameter estimation algorithm [11], but this approach needs to exact the carrier frequency before estimating the pseudo-random code. Some estimators based on cyclic spectral density function were proposed in [12,13], which has less computationally intensive, but the chip time width and carrier frequency should be known. In the above methods, only carrier frequency estimation is not enough, but the estimation of pseudo-random code requires prior knowledge or additional calculation, which requires time accumulation or multiple processing. Moreover, most of these methods have poor performance at low SNR and cannot be applied to the complex electromagnetic environment.

Because of the sensitivity to regular signals and immunity to noise under certain conditions, Duffing oscillators have often been used to detect weak signals [14,15,16,17]. The intermittent chaotic states in chaotic systems are known to enhance the practicability of weak signal detection in chaotic systems [18]. Recently, many methods for estimating the carrier frequency of BPSK signals using a Duffing oscillator have been developed [19,20], which can have great performance at very low SNR due to the Duffing oscillator. However, the pseudo-random sequence cannot be detected. The pseudo-random sequence estimation using the output characteristics of the Duffing oscillator excited by a known carrier frequency signal are studied in [21,22]. These methods are more easily to be calculated than the usual, but the carrier frequency of the BPSK signal at low SNR is not generally known under non-cooperative conditions. In short, these methods based on Duffing oscillator are limited in some way, but there have been few attempts to estimate both the carrier frequency and pseudo-random code of BPSK signals without prior knowledge using the Duffing oscillator.

In this paper, the relational expression among the state of Duffing oscillator, the phase code and the cosine function of the difference frequency in the intermittent chaotic state of the Duffing chaotic oscillator excited by BPSK signal is derived. Two parameter estimation methods for the joint estimation of carrier frequency and pseudo-random code based on this output characteristics are presented. These methods enable exact parameter estimations to be generated, even when no prior knowledge is available. Simulation and experiment results show that these methods have low computation complexity and high estimation precision, remain feasible in low SNR environments, and eliminate the high dependence of the traditional BPSK signal estimation method on the signal carrier frequency.

The remainder of paper is organized as follows. The next section analyzes the output characteristics of the BPSK signal in the Duffing oscillator system and deduces their relationship. In Section 3, the two blind parameter estimation methods are described. The simulation and experiment results in Section 4 demonstrate the feasibility of the proposed methods. Finally, the conclusions to this study are presented in Section 5.

## 2. Relationship among Functions in Duffing Oscillator System under Intermittent Chaotic State Excited by BPSK Signal

Traditional weak signal detection systems based on Duffing oscillator are excited by a standard sinusoidal signal, which can be expressed as

(1) $$\left\{ \begin{array}{l} \dot{x}=\omega y\\ \dot{y}=\omega (-ky+ax-bx^{3}+\gamma_{r}\cos(\omega t)+\gamma_{s}\cos((\omega +\Delta \omega )t+\varphi_{0})+n(t)) \end{array}\right.$$

where *x* is displacement, *k* is the damping coefficient, and −*ax*(*t*) + *bx*^3^(*t*) is the nonlinear restoring force. As shown in previous works, if we fix *k* = 0.5, *a* = *b* = 1, the Duffing oscillator system is more stable and represents better chaotic states [11,12,13,14]. *γ*_r_cos(*ωt*) is a periodic internal driving force with an amplitude of *γ*_r_ ≈ *γ*_c_, where *γ*_c_ is the critical threshold. *γ*_s_cos((*ω+*Δ*ω*)*t* + *φ*_0_) is the to-be-detected signal. *γ*_s_ is the amplitude, *φ*_0_ is the initial phase, and Δ*ω* is the frequency difference between the internal drive force signal and to-be-detected signal. *n*(*t*) is the stochastic disturbance, which is considered to be Gaussian white noise in this letter.

**Figure 1:**
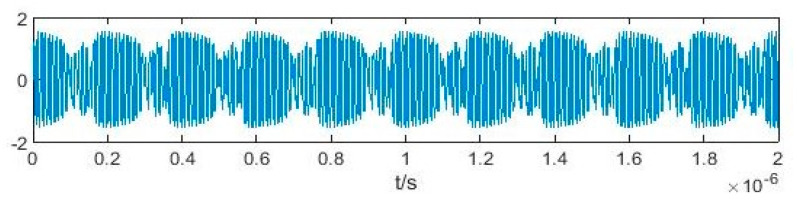
Output time domain waveform of the Duffing oscillator excited by a sinusoidal signal.

According to [18], the value of Δ*ω* can influence the equivalent driving force of the system, and then the chaotic states of Duffing oscillator can also change. If Δ*ω* = 0, after adding the signal to be measured, the system transforms from a chaotic state to a large-scale periodic state. If Δ*ω* ≠ 0, the system will be intermittently chaotic. When Δ*ω* ≤ 0.03*ω*, the intermittent chaotic state can be maintained regularly and stably. When Δ*ω* exceeds this limit, the intermittent chaotic state may be broken due to the insufficient maintenance time of the equivalent policy force. In weak signal detection, there is usually only a rough estimate for the frequency of the signal to be measured, which is often different from the drive signal frequency in the system. Thus, weak signal detection based on the intermittent chaotic state is universal and significant. Figure 1 illustrates the intermittent chaotic state of the Duffing oscillator excited by the standard sinusoidal signal. Here, *ω* = 100 MHz, Δ*ω* = 3 MHz, *γ*_r_ = 0.826, *γ*_s_= 0.1, and *φ*_0_ = 0. 

BPSK signals can realize phase modulation using pseudo-random codes containing some sequence of 1 s and −1 s. They can be expressed as

(2) $$s(t)=\gamma_{s}\cos((\omega +\Delta \omega )t+\varphi_{0}+\varphi_{i})$$

where *φ*_i_ = (0, *π*) is the phase code of the BPSK signal. Replacing the sinusoidal signal to be detected in Equation (1) with the BPSK signal, the new state equation of the Duffing oscillator is

(3) $$\left\{ \begin{array}{l} \dot{x}=\omega y\\ \dot{y}=\omega (-0.5y+x-x^{3}+\gamma_{r}\cos(\omega t)+\gamma_{s}\cos((\omega +\Delta \omega )t+\varphi_{0}+\varphi_{i})+n(t)) \end{array}\right.$$

**Figure 2:**
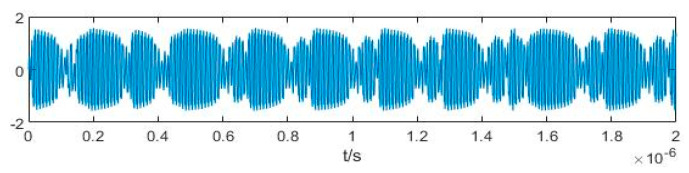
Output time domain waveform of the Duffing oscillator excited by binary phase shift keying (BPSK) signal.

Similar to the sinusoidal signal, BPSK signals can also cause the Duffing oscillator to transit intermittently between the chaotic state and large-scale periotic state if 0 < |Δ*ω*| ≤ 0.03*ω*. However, the duration of the different states is affected by both the difference frequency Δ*ω* and the phase code *φ*_i_. Hence, the Duffing oscillator does not have a stable and regular intermittent chaotic period, unlike for the sinusoidal signal. The state in this condition is illustrated in Figure 2. Like the sinusoidal signal, we also fix *ω* = 100 MHz, Δ*ω* = 3 MHz, *γ*_r_ = 0.826, *γ*_s_ = 0.1, and *φ*_0_ = 0.

To estimate the parameters of the BPSK signal, we need further analysis of the output characteristics of the Duffing oscillator. For Equation (3), the equivalent driving force of the system can be written as *γ*_e_cos(*ωt* + *θ*), where

(4) $$\gamma_{e}(t)=\sqrt{\gamma_{r}^{2}+2\gamma_{r}\gamma_{s}\cos(\Delta \omega t+\varphi (t))+\gamma_{s}^{2}}$$

(5) $$\theta (t)=\arctan\left[\frac{\gamma_{s}\sin(\Delta \omega t+\varphi (t))}{\gamma_{r}+\gamma_{s}\cos(\Delta \omega t+\varphi (t))}\right]$$

Here, *φ*(*t*) = *φ*_0_ + *φ*_i_. Because the to-be-detected signal is weak, so *γ*_s_ ≈ 0, *γ*_r_ ≈ *γ*_c_, and *γ*_s_ << *γ*_r_, the value of *θ*(*t*) can be neglected. Therefore, the *γ*_e_(*t*) can be written as

(6) $$\gamma_{e}(t)\approx \sqrt{\gamma_{c}^{2}+2\gamma_{c}\gamma_{s}\cos(\Delta \omega t+\varphi (t))}$$

According Equation (6), we can get

(7) $$\mathrm{sgn}(\gamma_{e}(t)-\gamma_{c})=\mathrm{sgn}(\cos(\Delta \omega t+\varphi (t)))$$

Here, sgn(*number*) function represents the symbolic sign function: if *number* > 0, sgn = 1; if *number* = 0, sgn = 0; if *number* < 0, sgn = −1. The relationship among the values of sgn function, *φ*_i_, and the time *t* is presented in Table 1. In this table, *t*_1_ = (2*kπ* − *π*/2 − *φ*_0_)/|Δ*ω*|, *t*_2_ = (2*kπ*+*π*/2 − *φ*_0_)/|Δ*ω*|, *k* = 0, 1, 2.

According to Equation (7) and Table 1, although the intermittent chaotic state is not regular, it has some implicit periodicity, and the relationship among the state of the system at some point, the phase code *φ*_i_ and the cosine of the difference frequency cos(Δ*ωt* + *φ*_0_) can be expressed as

(8) $$\mathrm{sgn}(\gamma_{e}(t)-\gamma_{c})=\mathrm{sgn}(\cos(\Delta \omega t+\varphi_{0}))\times \cos(\varphi_{i})$$

If the system is in the large-scale periodic state, $\gamma_{e}(t)>\gamma_{c}$, sgn($\gamma_{e}(t)>\gamma_{c}$) = 1; if it is in the chaotic state or critical state, $\gamma_{e}(t)\leq \gamma_{c}$, and the value of sgn($\gamma_{e}(t)\leq \gamma_{c}$) is −1 or 0. Thus, we can define S*ys*(*t*) as the state of Duffing oscillator:

(9) $$Sys(t)=\left\{ \begin{array}{ll} 1, & \mathrm{sgn}(\gamma_{e}(t)-\gamma_{c})=1\\ -1, & \mathrm{else} \end{array}\right.$$

We fix D*f*(*t*) as the output state of the difference frequency when the input is the sinusoidal signal, that is

(10) $$\begin{array}{ll} Df(t) & =\{\begin{array}{cc} 1, & \mathrm{sgn}(\cos(\Delta \omega t+\varphi_{0}))=1\\ -1, & \mathrm{else} \end{array}\\ & =\{\begin{array}{cc} 1, & t_{1}<t<t_{2}\\ -1, & \mathrm{else} \end{array} \end{array}$$

The phase code can be represented as

(11) $$Pc(t)=\{\begin{array}{c} 1\\ -1 \end{array}\begin{array}{c} ,\\ , \end{array}\begin{array}{c} \cos(\varphi_{i})=1\\ \cos(\varphi_{i})=-1 \end{array}$$

According to Equation (8), the relationship of these functions can be written as

(12) Sys(t)=Df(t)×Pc(t)

As S*ys*(*t*), D*f*(*t*) and P*c*(*t*) are all bi-valued functions with outputs of 1 or –1, if two of these three functions are known, the third can be determined by a simple multiplication. The equivalent forms of Equation (12) are

(13) Df(t)=Sys(t)×Pc(t)

(14) Pc(t)=Sys(t)×Df(t)

**Figure 3:**
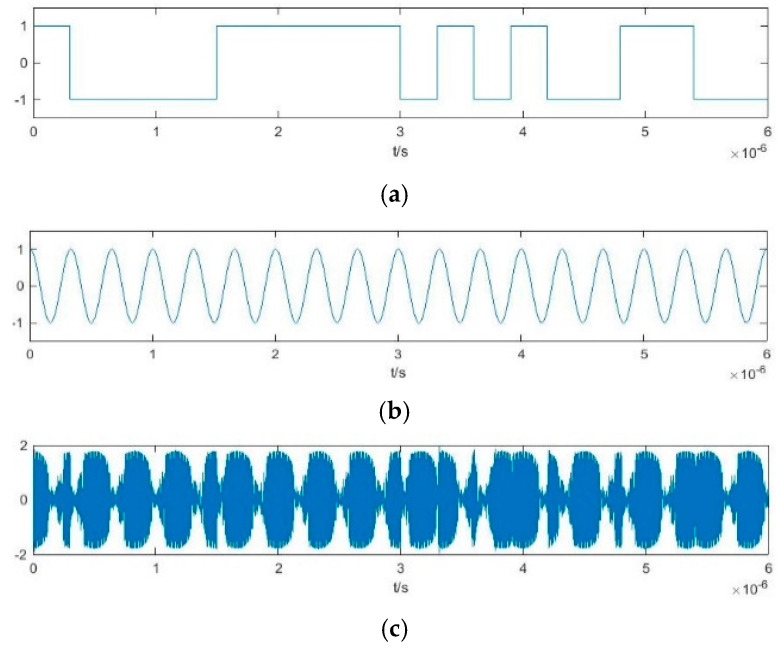
Time-domain waveforms of (**a**) *φ*_i_; (**b**) cos(Δ*ωt* + *φ*_0_); (**c**) *y*(*t*).

**Figure 4:**
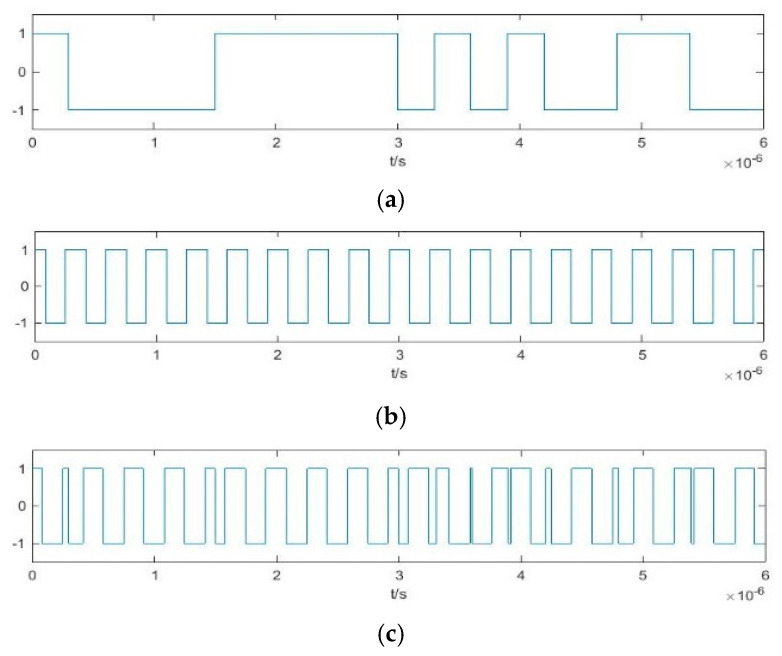
Time-domain waveforms of (**a**) P*c*(*t*); (**b**) D*f*(*t*); (**c**) S*ys*(*t*).

We wish to simulate an X-band radar BPSK signal with *ω +* Δ*ω* = 10 GHz; in this case, the data volume is very large and requires significant computation. Hence, we set *ω +* Δ*ω* = 100 MHz (which can be regarded as a radar BPSK signal after down-conversion), increase the calculation speed by a factor of 100, and set Δ*ω* = 3 MHz, *φ*_0_ = 0. The pseudo-random sequence is m sequence with the symbol width of 300 ns. It can be represented as 10000111110101001100 over a period of 6 × 10^−6^ s. Then, we obtain (a) *φ*_i_, (b) cos(Δ*ωt* + *φ*_0_) and (c) the output of Duffing oscillator *y*(*t*) in Figure 3. After binarization of the values 1 and –1, Figure 4 presents the time-domain waveforms of (a) P*c*(*t*), (b) D*f*(*t*), and (c) S*ys*(*t*). This figure provides a visual representation of the multiplicative relation in Equation (12).

This relationship provides a theoretical basis for blind parameter estimation of BPSK signal. In actual applications, we can easily find S*ys*(*t*) from the Duffing oscillator. Therefore, the key to parameter estimation is to obtain D*f*(*t*) or P*c*(*t*). If we can find either one of them, we can also determine the other based on Equations (12)–(14). On the basis of this theory prerequisite, we propose two estimation methods in the next section.

## 3. Parameter Estimation Method for BPSK Signals Based on Output Characteristics Including Implied Periodicity and Array Synchronization of Duffing Oscillator

In previous articles, BPSK signal detection and parameter estimation methods based on the Duffing oscillator have used special signals to increase the known information. However, these approaches require preprocessing to obtain more a priori information, which affects the real-time capability of the reconnaissance system, and are not applicable in all cases. Therefore, completing the blind estimation of the BPSK signal with less prior information, sometimes when only S*ys*(*t*) is known, is the focus of this study.

### 3.1. Parameter Estimation Method for BPSK Signals Based on Implied Periodicity

**Figure 5:**
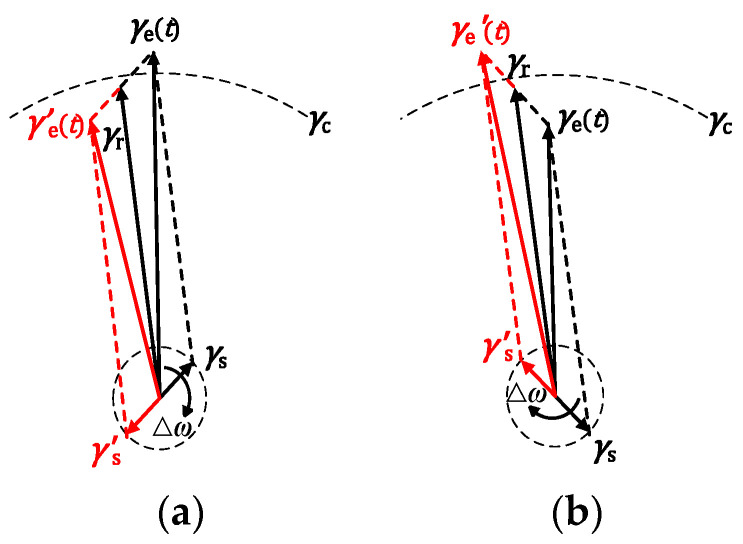
Vector diagram of amplitude of equivalent driving force under the action of BPSK signal: (**a**) before the change, *γ*_e_ > *γ*_c_; (**b**) before the change, *γ*_e_ < *γ*_c_.

The output of Duffing oscillator excited by BPSK signal is affected by difference frequency Δ*ω* and phase code *φ*_i_. The vector diagram of driving forces of the Duffing oscillator under the influence of BPSK signal is shown in Figure 5. The *γ*_e_ changes alternately periodically based on Δ*ω*. When *φ*_i_ is converted from 0 to π, *γ*_s_ is transferred to *γ*_s_^’^, which causes the changes of numerical value relation between the new equivalent drive force *γ*_e_^’^(*t*) and threshold *γ*_c_; that is the reason why the intermittent chaotic state changes in this system.

**Figure 6:**
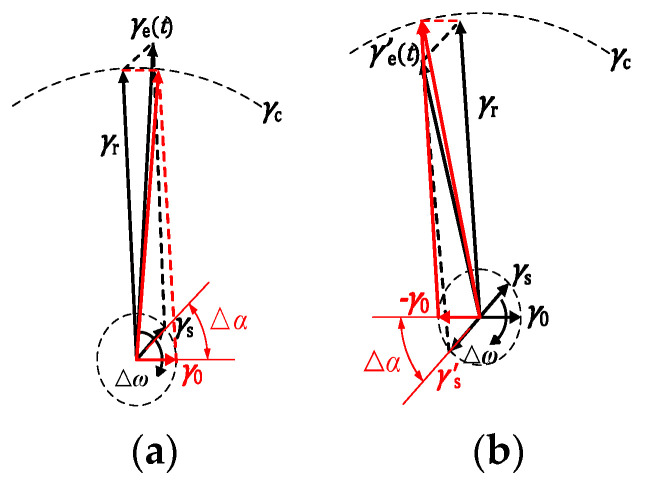
Vector diagram of movement rule under the action of BPSK signal: (**a**) before the change of *φ*_i_; (**b**) after the change of *φ*_i_.

Taking the case of Figure 5a as an example, letting the value of *γ*_s_ to be *γ*_0_ when *γ*_s_ = *γ*_c_, we can get the vector diagram in Figure 6 by studying the moment of system state transition. As shown in Figure 6a, before the change of the value of *φ*_i_, *γ*_e_ > *γ*_c_, the system is in large-scale motion; the value of *γ*_e_ will become smaller as it rotates clockwise, and after the time of Δ*α*/Δ*ω*, *γ*_e_ = *γ*_c_, the system will move to a chaotic motion. In Figure 6b, after the change of *φ*_i_, *γ*_e_^’^ *<* *γ*_c_, the system is inchaotic motion; while after the time of Δ*α*/Δ*ω*, and it will become large scale motion. In summary, the state of Duffing oscillator system will transform before and after the conversion of *φ*_i_, but it does not break the periodic vector motion of the system, the system will still change its state at the same time as the Duffing oscillator driven by the sinusoidal signal after the time of Δ*α*/Δ*ω*. According to these, although the intermittent chaotic state is not regular, it also has implicit periodicity.

**Figure 7:**
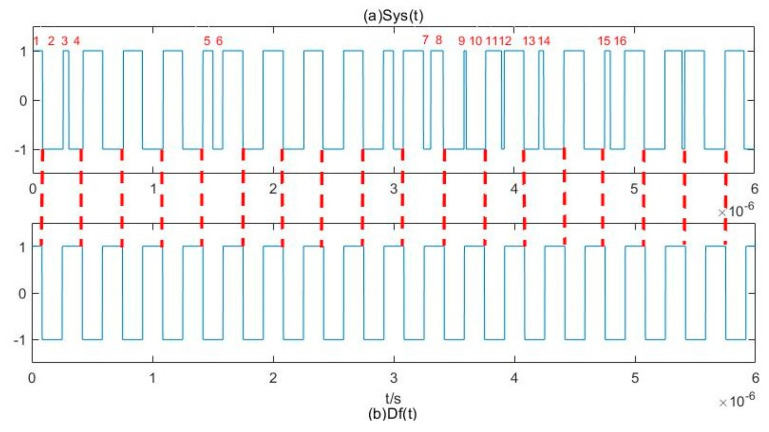
Numerical variation relationship of S*ys*(*t*) and D*f*(*t*).

**Figure 8:**
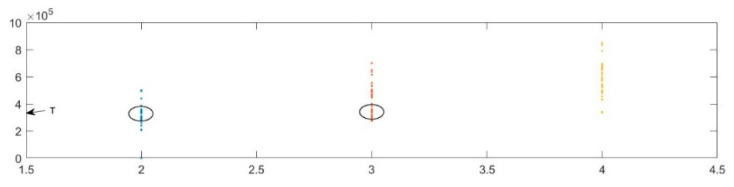
The points of adjacent segments.

Figure 7 compares the numerical variation of S*ys*(*t*) and D*f*(*t*). The length between the adjacent red fine line is a period *T* of D*f*(*t*). No direct periodicity can be observed in S*ys*(*t*), whereas the seemingly irregular numerical change implies the periodicity of D*f*(*t*). The length of different segments in S*ys*(*t*) can be marked as 1~16 (the same lengths are ignored), and the sum of 2 + 3 + 4, 2 + 5 + 6, 2 + 7 + 8, 2 + 9 + 10, 2 + 11 + 12, 2 + 13 + 14, 2 + 15 + 16 (where, for example, 2 refers to all segments with lengths equal to the segment marked as 2) in this figure is exactly the period of D*f*(*t*). Thus, we first calculate the length of time that S*ys*(*t*) spends in the 1 or –1 states, then calculate the sum of lengths of every two and three adjacent segments (*T*_2i_ and *T*_3i_, respectively) and find their minimum *T*_3min_. Some of these summed lengths of adjacent segments will be less than the period *T* of D*f*(*t*), and some of them will exceed *T*. Statistically, however, it is more likely that the summed lengths of these segments will approximate the period *T* (we set Δ*ω* = 2π × 3 MHz, so the points of *T* is 3.333 × 10^−7^ s), see Figure 8. Therefore, the period *T* of D*f*(*t*) can be obtained by summing the time length and using statistical methods. In this paper, we discard values of *T*_2i_ that are less than 0.9 × *T*_3min_ and obtain the period *T* of D*f*(*t*) by averaging the remaining *T*_2i_.

Assuming Δ*ω* > 0, the frequency difference can be obtained as Δ*ω* = 2π/*T*. A binary signal D*f*_0_(*t*) with the same period *T* can be constructed:

(15) $$Df_{0}(t)=\{\begin{array}{c} 1\\ –1 \end{array}\begin{array}{c} ,\\ , \end{array}\begin{array}{c} \mathrm{sgn}(\cos(\Delta \omega t))=1\\ \mathrm{else} \end{array}$$

From Equations (10) and (15) we can see D*f*(*t*) and D*f*_0_(*t*) differ by an initial phase *φ*_0_. To eliminate all the numerical jumps in S*ys*(*t*) caused by D*f*_0_(*t*), it is necessary to find and compensate this initial phase *φ*_0_. We find the position in S*ys*(*t*) for which the length of the two adjacent segments is *T*, select one of these segments, and set its starting point as *t*_0_. Taking this point as a reference, then *φ*_0_ satisfies

(16) $$\varphi_{0}=k\times \frac{T}{2}-t_{0}$$

where *k* is an arbitrary integer. Taking an appropriate value of *k* to shift D*f*_0_(*t*) to the right by *φ*_0_, the reconstructed signal D*f*_rc_(*t*) can be obtained. We can then replace D*f*(*t*) with D*f*_rc_(*t*) and obtain P*c*_rc_(*t*) (the estimation of P*c*(*t*)).

In the above process, we assume Δ*ω* > 0. However, if we want to determine the accurate carrier frequency, we need to make a judgment about the sign of Δ*ω*. In this letter, a simultaneous pseudo-random sequence estimation method for multiplex Duffing oscillators with different internal driving force is applied. Taking two channels as an example, two difference frequencies |Δ*ω*_1_*|* and |Δ*ω*_2_*|* can be obtained. By comparing their value and the different frequencies of the two internal driving forces, the positive and negative values of Δ*ω*_1_ and Δ*ω*_2_ can be determined, and then two estimated values of carrier frequency (*ω* + Δ*ω*_1_ and *ω* + Δ*ω*_2_) can be obtained. Averaging these values gives the estimated carrier frequency of the BPSK signal. The estimation accuracy of the pseudo-random sequence can be improved by increasing the number of oscillators.

**Figure 9:**
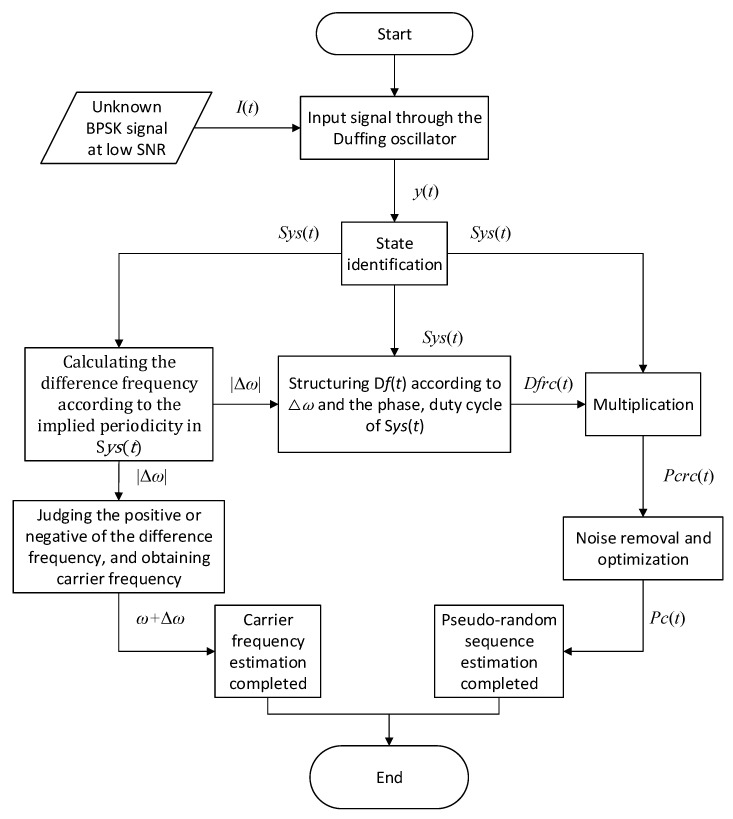
Flow diagram of the parameter estimation method based on the implied periodicity.

**Figure 10:**
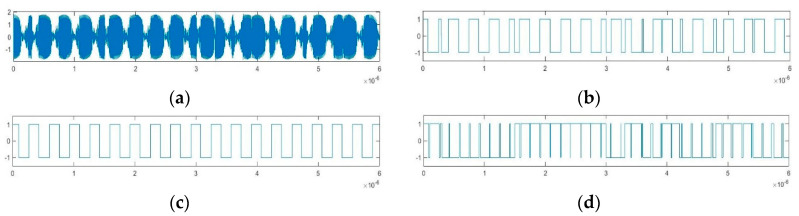
Time-domain graph of key nodes in pseudo-random sequence estimation based on implied periodicity: (**a**) *y*(*t*); (**b**) S*ys*(*t*); (**c**) D*f*_rc_(*t*); (**d**) Pc_rc_(*t*).

The estimation method for the pseudo-random sequence and carrier frequency of the BPSK signal based on implied periodicity of Duffing oscillator is illustrated in Figure 9. As shown in Figure 9, we can get S*ys*(*t*) after binarization of the state identification of Duffing oscillator excited by the unknown BPSK signal. According to S*ys*(*t*), Δ*ω* can be obtained by the implied periodicity of Duffing oscillator, and then the carrier frequency can be estimated after judging the plus-minus sign of Δ*ω*. Besides, based on Δ*ω* and the phase, duty cycle of S*ys*(*t*), we can reconstruct D*f*(*t*). Finally, the pseudo-random sequence can be estimated based on the multiplication formula in Equation (14). This approach is used for the pseudo-random sequence estimation of BPSK signals in Figure 3. The time-domain waveform of several key nodes in the process is shown in Figure 10. Figure 10a shows the output of the Duffing oscillator system under the action of BPSK signal; Figure 10b is the binarized output S*ys*(*t*); Figure 10c is the reconstructed output state of frequency difference named D*f*_rc_(*t*) based on this method; and Figure 10d is the estimated result, P*c*_rc_(t), given by this method. 

**Figure 11:**
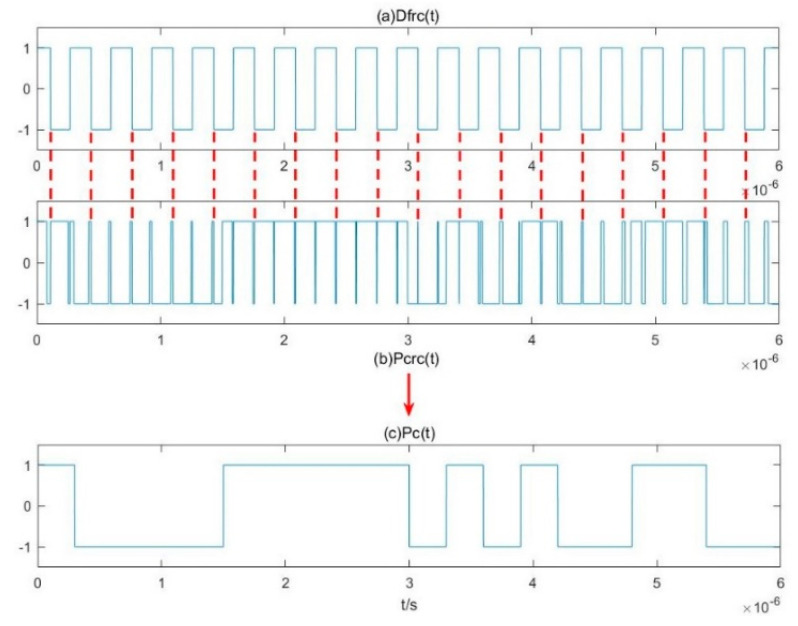
Deburring method of P*c*_rc_(*t*).

A deburring method for P*c_rc_*(*t*) is now studied. The burrs in P*c*_rc_(*t*) are mainly caused by the small errors of the reconstructed signal D*f*_rc_(*t*) and the binary system output S*ys*(*t*) for the same intermittent chaotic state. Compared with the time-domain waveform diagrams of P*c*_rc_(*t*) and D*f*_rc_(*t*), almost all the burrs in P*c*_rc_(*t*) correspond to a state transition of D*f*_rc_(*t*). Therefore, as shown in Figure 11, for each state transition moment of D*f*_rc_(*t*), if P*c*_rc_(*t*) has a pair of state transitions in a relatively short time around this moment, the two-state transitions are eliminated at the same time, resulting in pseudo-random sequences without burrs. It is clear that the sequence is 10000111110101001100, which is consistent with the pseudo-random sequence given in Figure 3. 

For carrier frequency estimation, two-channel Duffing oscillators are adopted with internal driving force signal frequencies of 98 MHz and 103 MHz, respectively. The measured frequency difference of oscillator 1 is 1.99854 MHz, and that of oscillator 2 is 2.99814 MHz. According to the numerical relation, the signal to be tested should be between the frequencies of oscillator 1 and oscillator 2. The estimated value of the carrier frequency is the average of the estimated values of the two oscillators, which in this case is 100.0002 MHz (oscillator 1: 99.99854 MHz, oscillator 2: 100.00186 MHz), an error of only 0.0002%. According to the results, the parameter estimation method for BPSK signals based on implied periodicity can realize high accurate blind estimation for a carrier frequency and pseudo-random sequence.

### 3.2. Parameter Estimation Method for BPSK Signals Based on Pilot Frequency Array Synchronization

In a weak signal detection system, the use of a Duffing oscillator array is of great importance in many ways. By setting different internal driving force signals for each Duffing oscillator, the array can achieve multiply frequency measuring ranges and increased detection and estimation precision while eliminating the phase and frequency of blind areas.

When BPSK signals pass the Duffing oscillator array with different internal driving force signals, each oscillator output corresponding to different intermittent chaos period. However, all the oscillators are controlled by the same phase code *φ*_i_, the states of them will change at the same time. Therefore, the intermittent chaos states of array have a certain regularity, which can be used for the blind estimation of pseudo random sequences.

For each Duffing oscillator array, the internal driving force signal is $\gamma_{r}\cos(\omega_{j}t)$. At this time, the binarization cosine function of the frequency difference between the internal driving force signal and the BPSK signal D*f*(*t*) varies with the frequency of the internal driving force signal

(17) $$Df_{j}(t)=\{\begin{array}{c} 1\\ -1 \end{array}\begin{array}{c} ,\\ , \end{array}\begin{array}{c} \mathrm{sgn}(\cos(\Delta \omega_{j}t+\varphi_{0}))=1\\ \mathrm{else} \end{array}$$

**Figure 12:**
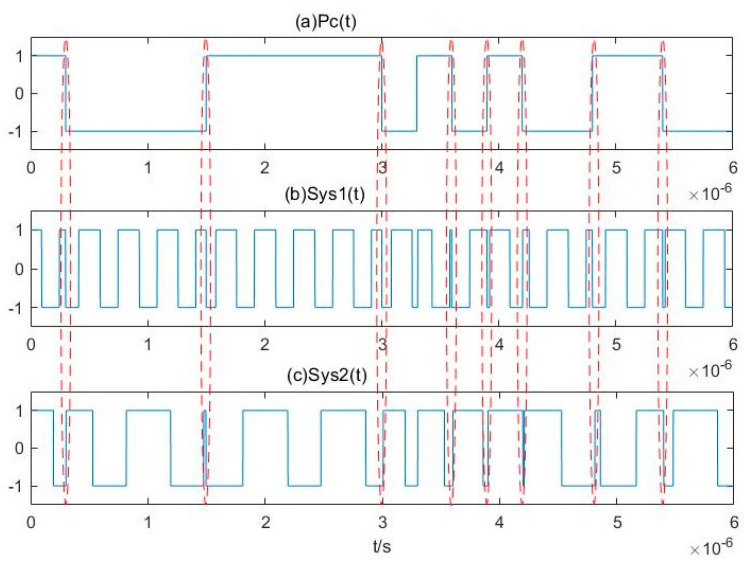
Pilot frequency array synchronization of the output characteristics of Duffing oscillator.

After inputting the BPSK signal, the output characteristics are converted into binary S*ys*_j_(*t*). Taking a simple array of two Duffing oscillators as an example, the binarization of the output characteristics S*ys*_1_(*t*) and S*ys*_2_(*t*) involves numerical conversion between 1 and –1 simultaneously affected by P*c*(*t*), D*f*_1_(*t*), and D*f*_2_(*t*). Due to the difference in the period of D*f*_1_(*t*) and D*f*_2_(*t*), the time-domain waveforms of S*ys*_1_(*t*) and S*ys*_2_(*t*) are quite different. However, as the time of the output state transformation caused by P*c*(*t*) in each Duffing oscillator in the array is always the same, there will always be a time when the numerical transformation occurs synchronously in S*ys*_1_(*t*) and S*ys*_2_(*t*). This time corresponds to the numerical transformation in P*c*(*t*), as shown in Figure 12. Figure 12a is the pseudo-random sequence P*c*(*t*). Figure 12b,c show the binarization results of S*ys*_1_(*t*) and S*ys*_2_(*t*) obtained after the BPSK signal modulated by P*c*(*t*) has passed through two different Duffing oscillators in the array. The synchronous numerical transformations are marked with red ellipses, and these are consistent with the jump law of P*c*(*t*). This constitutes the array synchronization of the output characteristics of the Duffing oscillator for the BPSK signals. The array synchronization obtained through the Duffing oscillator array with different frequencies is called pilot frequency array synchronization.

According to this property, we can input the BPSK signal into a group of Duffing oscillator arrays that have different internal driving force signals with different frequencies. After obtaining the binarization output characteristics, we can determine the position at which the numerical conversion takes place synchronously according to the array synchronization and obtain P*c*(*t*). Subsequently, we can calculate the D*f*_i_(*t*) of the array according to Equation (12). Averaging D*f*_i_(*t*), we then obtain the estimated carrier frequency D*f*(*t*). For a pilot frequency Duffing oscillator array, because the frequency range of a single oscillator is about 0.03 *ω*, we set the frequency difference between each oscillator and the center frequency *ω* to be less than 0.03 *ω*.

**Figure 13:**
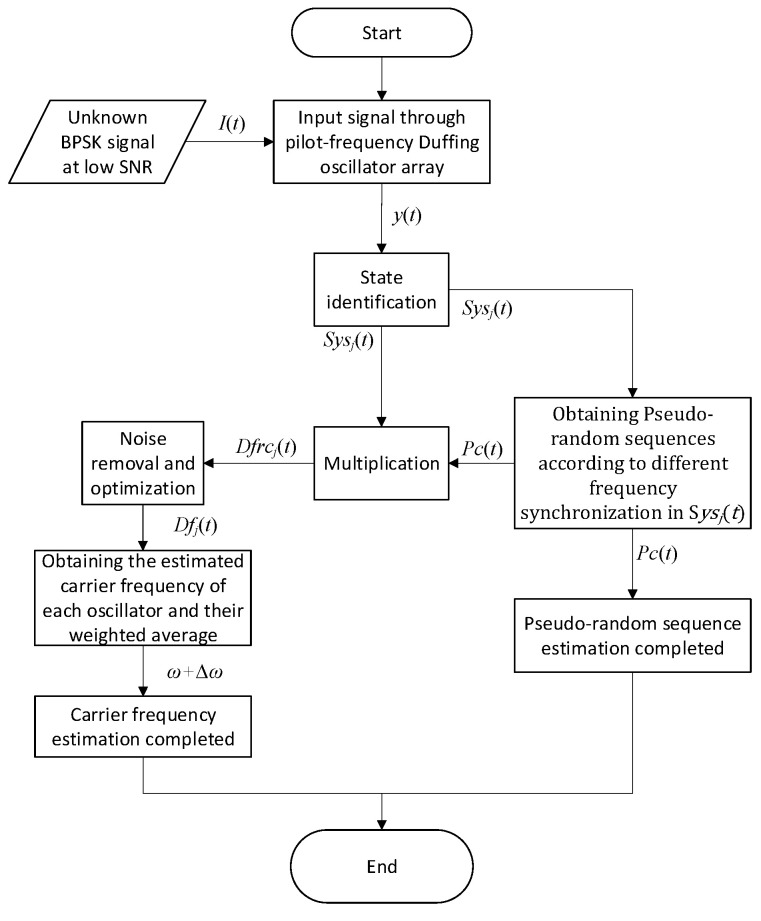
Flow diagram of the parameter estimation method for BPSK signal based on pilot frequency array synchronization of the Duffing oscillator.

We take the pilot frequency Duffing array as an example and propose a parameter estimation method for BPSK signals based on the synchronization of the array, as shown in Figure 13. As in Figure 9, we can get S*ys*_j_(*t*) after binaryzation of the state identification of Duffing oscillator array excited by the unknown BPSK signal. Then the pseudo-random sequence can be estimated by the pilot frequency array synchronization of Duffing oscillators. After obtaining D*f*_i_(*t*) based on the multiplication formula in Equation (13), we can complete the estimation of the carrier frequency.

**Figure 14:**
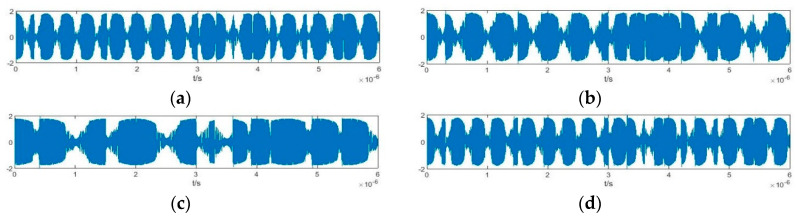
Output of the Duffing oscillator array: (**a**) oscillator 1; (**b**) oscillator 2; (**c**) oscillator 3; (**d**) oscillator 4.

According to the parameter estimation method in Figure 13, the carrier frequency and pseudo-random sequence of the BPSK signal in Figure 3 can be estimated. The signal to be detected is processed through an array formed by four Duffing oscillators with internal driving force frequencies of 97 MHz (oscillator 1), 98 MHz (oscillator 2), 101 MHz (oscillator 3), and 103 MHz (oscillator 4). The output of this system is shown in Figure 14.

**Figure 15:**
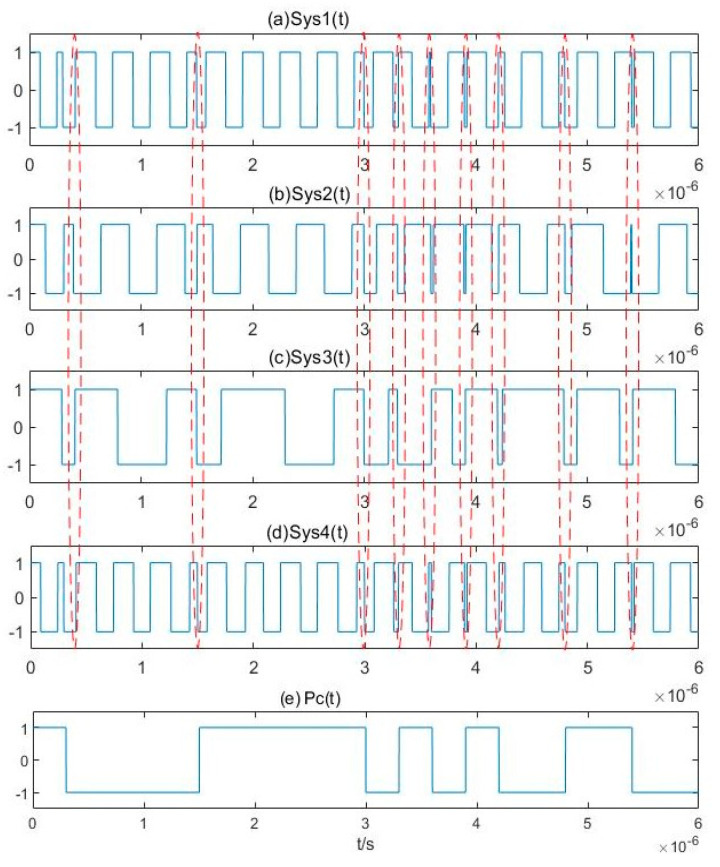
Output of the binarized Duffing oscillator array and estimation results of the pseudo-random sequence.

After binarizing this array, the pseudo-random sequence estimation can be obtained based on pilot frequency synchronization. The results are shown in Figure 15.

**Figure 16:**
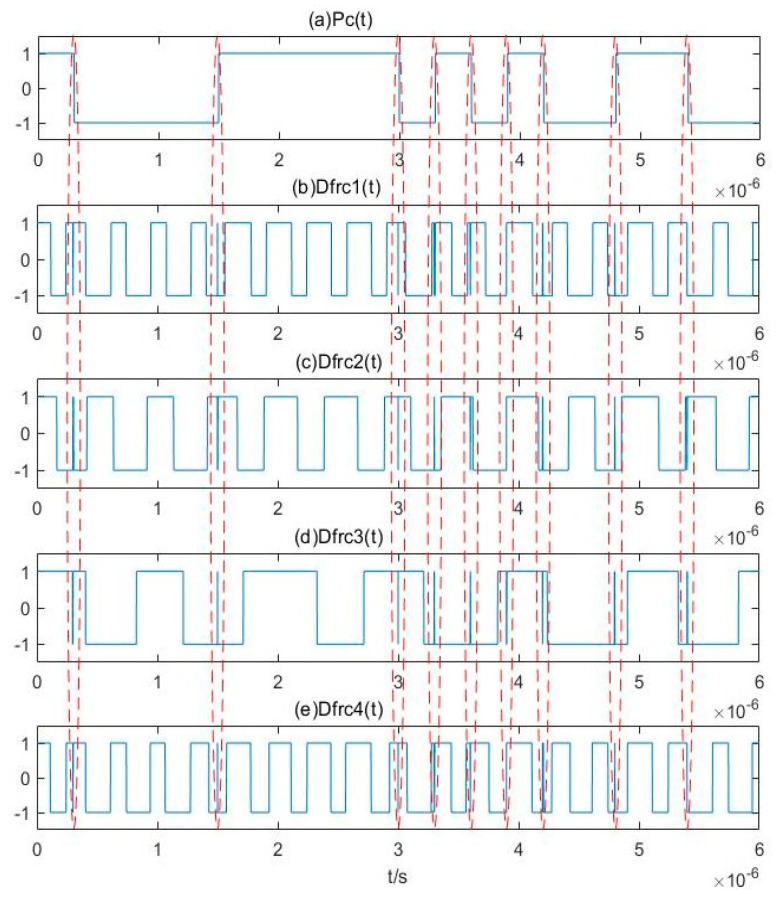
Cosine function of binarized difference frequency of Duffing oscillator array.

**Figure 17:**
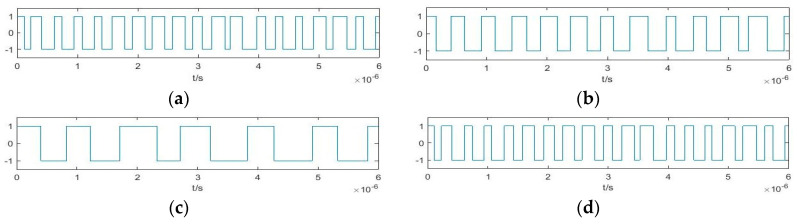
Estimated results after deburring: (**a**) D*f*_1_(*t*); (**b**) D*f*_2_(*t*); (**c**) D*f*_3_(*t*); (**d**) D*f*_4_(*t*).

The cosine function of binarized frequency difference obtained from the Duffing oscillator array according to Equation (12) is shown in Figure 16. As for the analysis of Figure 11, the arrows in this figure represent the time at which the value of the pseudo-random sequence shown in Figure 15e changes. If two state changes occur in the vicinity of this time within a relatively short period, they will be eliminated at the same time, and the estimated results after deburring can be obtained, as shown in Figure 17.

The frequencies of four Duffing oscillators as shown in Figure 17. The frequency differences between Duffing oscillators 1~4 and the center frequency are approximately 3.02816 MHz, 2.02925 MHz, 1.02999 MHz, and 3.03061 MHz, respectively. Therefore, the estimated center frequency based on these four oscillators are 100.02816 MHz, 100.02925 MHz, 99.97001 MHz, and 99.96939 MHz. The average value is 99.9992 MHz, with an error of only 0.0008%. These results show that the proposed method can estimate the signal parameters effectively.

## 4. Experimental Validation Using the BPSK Signal Parameter Estimation Method

### 4.1. Simulation Experiment

To verify the feasibility of the two parameter estimation methods for BPSK signal, their performance in a Duffing oscillator weak signal detection system needs to be investigated. Thus, experiments were conducted to examine the estimation accuracy of the carrier frequency and the similarity of pseudo-random sequences under various SNRs. As a performance reference, the pseudo-random sequence was also estimated directly based on the known carrier frequency.

As in the simulation described in Section 3, the carrier frequency of the to-be-detected BPSK signal is 100 MHz, the symbol width of pseudo-random sequence is 300 ns, and signal amplitude is 0.6. The two-way Duffing oscillators adopted for the Duffing oscillator array based on implied periodicity have internal driving force frequencies of 103 MHz and 98 MHz. The Duffing oscillator array based on pilot frequency array synchronism is composed of four different Duffing oscillators with frequencies of 97 MHz, 98 MHz, 101 MHz, and 103 MHz, respectively. For the Duffing oscillator with the known carrier frequency, the driving force frequency is 100 MHz. The dynamic amplitudes of all dynamic amplitudes of Duffing oscillators are set to be close to the critical value, that is, *γ*_r_ = 0.826. These parameters are shown in Table 2.

**Figure 18:**
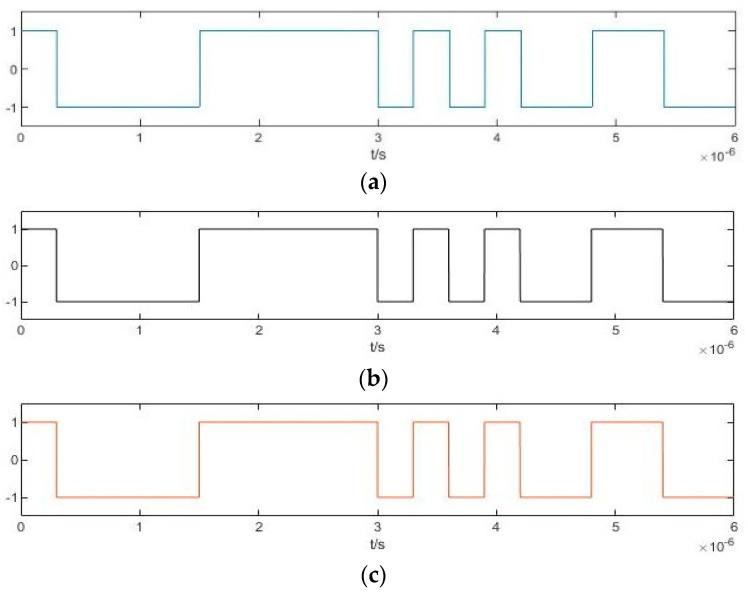
Pseudo-random sequence estimation results based on three methods with SNR = –10 dB: (**a**) based on implied periodicity; (**b**) based on pilot frequency array synchronization; (**c**) based on known carrier frequency.

**Figure 19:**
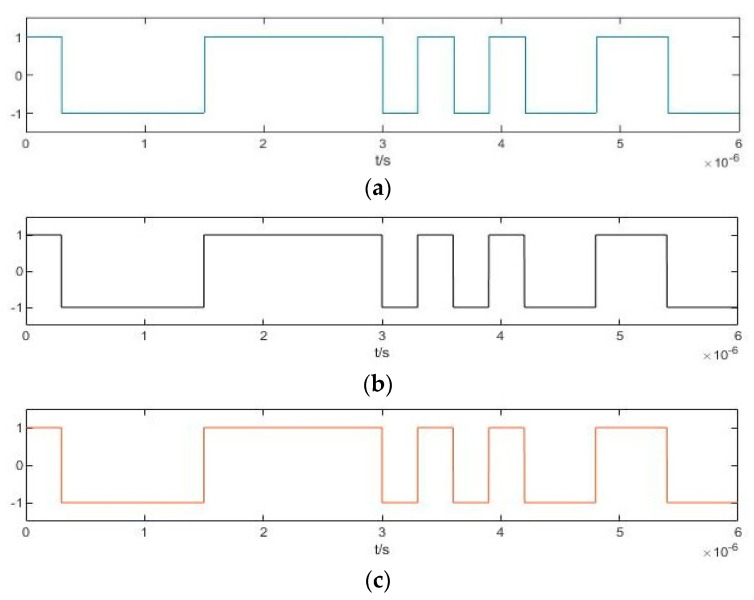
Pseudo-random sequence estimation results based on three methods with signal-to-noise ratio (SNR) = –20 dB: (**a**) based on implied periodicity; (**b**) based on pilot frequency array synchronization; (**c**) based on known carrier frequency.

**Figure 20:**
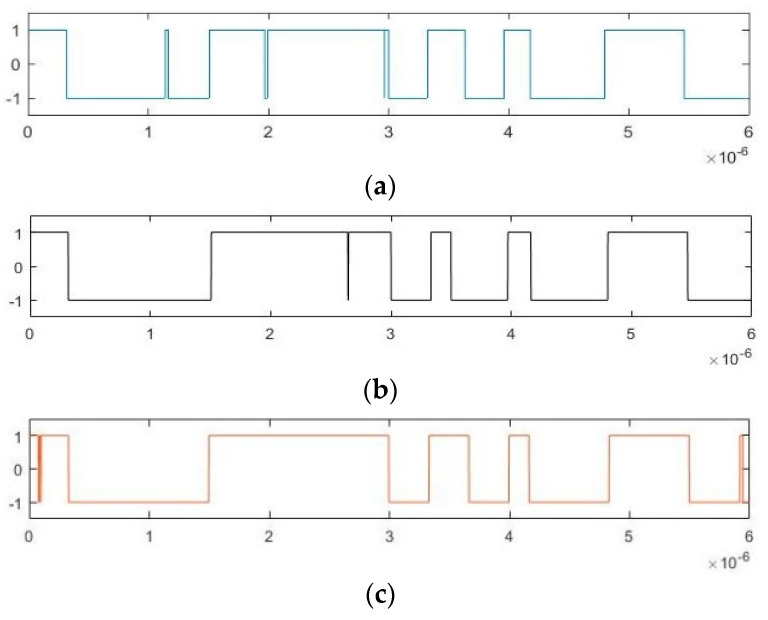
Pseudo-random sequence estimation results based on three methods with SNR = −30 dB: (**a**) based on implied periodicity; (**b**) based on pilot frequency array synchronization; (**c**) based on known carrier frequency.

**Figure 21:**
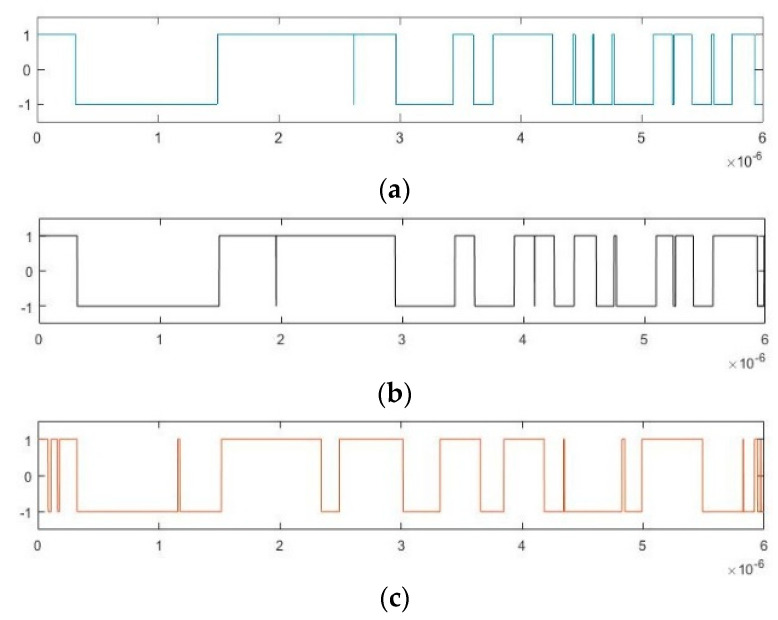
Pseudo-random sequence estimation results based on three methods with SNR = −35 dB: (**a**) based on implied periodicity; (**b**) based on pilot frequency array synchronization; (**c**) based on known carrier frequency.

BPSK signal detection under different SNR is realized by adjusting Gaussian noise variance σ^2^. To produce SNR = −10 dB, −20 dB, −30 dB, and −35 dB, we set σ^2^ to be 1.8, 18, 180, and 569.21, respectively. Under such conditions, the pseudo-random sequences obtained by the three methods are shown in Figure 18, Figure 19, Figure 20 and Figure 21. According to Figure 18 and Figure 19, when the SNR is −10 dB or −20 dB, the three methods all obtain good pseudo-random sequence estimation results. As SNR decreases, the difference between the chaotic state and the large-scale periodic state becomes smaller, and the identification becomes more difficult. Figure 20 and Figure 21 show the gradual appearance of several burrs that are difficult to remove. However, although the carrier frequency of the BPSK signal is unknown, the accuracy of the two blind estimation methods proposed in this paper approaches that of the traditional parameter estimation method with known carrier frequency. The pseudo-random sequence estimation also achieves good accuracy under the −35 dB SNR.

In parameter estimation, the correlation similarity coefficient is widely used to characterize the estimation accuracy of the pseudo-random sequence. This is the ratio of the peak value of the cross-correlation function between the obtained pseudo-random sequence and the original sequence to the peak value of the autocorrelation function of the original sequence. Table 3 presents the correlation similarity coefficients between the obtained pseudo-random sequences and the original sequences estimated by the three methods at different SNR. The pseudo-random sequences estimated by the three methods under −35 dB all have good cross-correlation performance with respect to the original sequence (correlation similarity coefficient >0.9). Relatively speaking, when the SNR is relatively high, the BPSK signal parameter estimation method based on array synchronization is closer to (and sometimes even better than) the estimation result of the special case in which the carrier frequency is known. When SNR is less than −35 dB, the correlation similarity coefficients obtained by the three methods decrease and take similar values.

The carrier frequency estimation results of the two parameter estimation methods proposed in the paper at different SNR are shown in Table 4. Under an SNR of −35 dB, the parameter estimation methods based on implicit periodicity and array synchronization achieve high precision. Parameter estimation method based on the implicit periodicity produces more accurate carrier frequency estimations. This probably because the implicit periodicity method estimates carrier frequency directly, whereas array synchronization first estimates the pseudo-random sequence, and then performs additional multiplication operations to get the carrier frequency, which will produce more errors. 

The simulation results show that the two parameter estimation methods proposed in this paper for the detection of weak BPSK signals based on the Duffing oscillator system can achieve high precision under −35 dB SNR. Under the condition of an unknown carrier frequency, these methods achieve similar estimation precision to the traditional approach in which the carrier frequency is known.

### 4.2. Semi-physical Simulation Experiment

**Figure 22:**
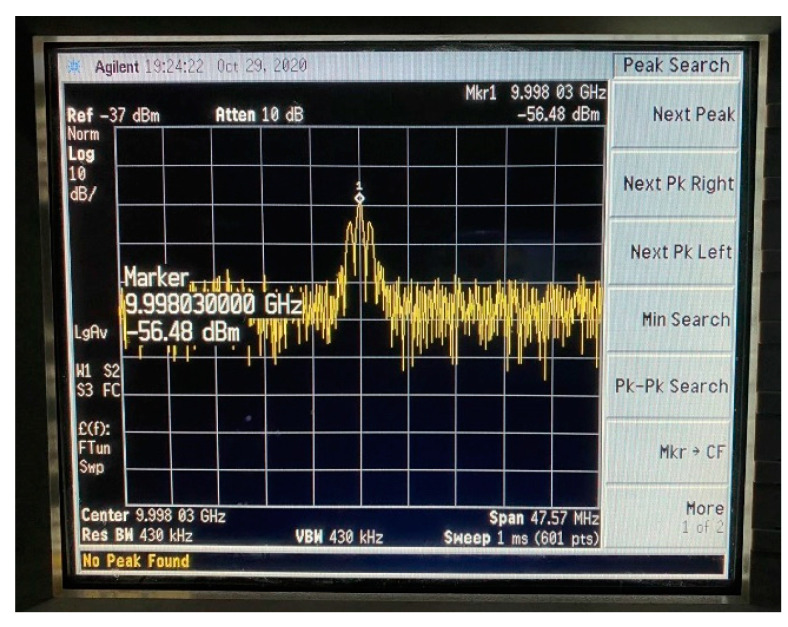
Spectrum diagram of emitted BPSK signal.

**Figure 23:**
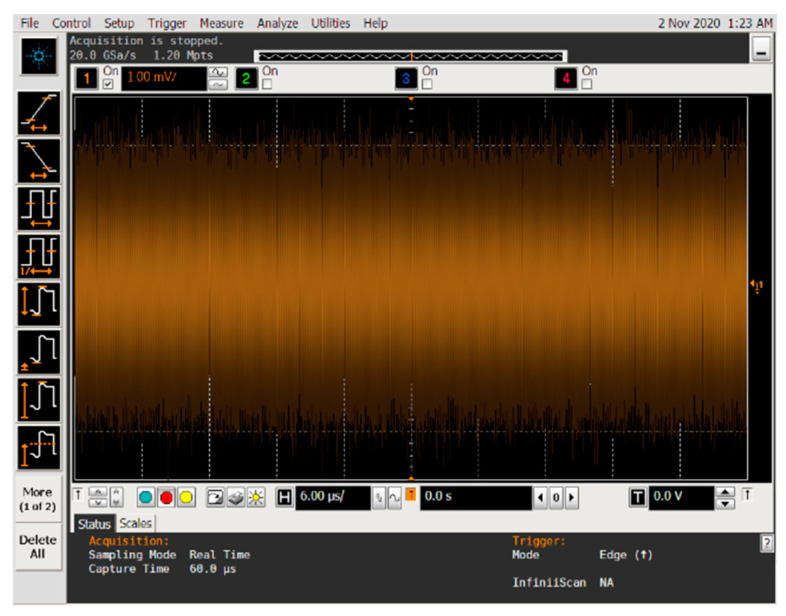
Time-domain diagram of emitted BPSK signal after down-conversion.

**Figure 24:**
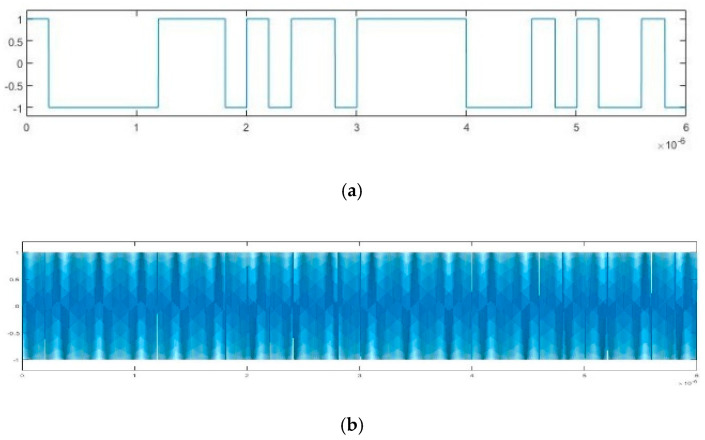
Estimated result based on Duffing oscillator implied periodicity: (**a**) pseudo-random sequence; (**b**) reconstructed BPSK signal.

**Figure 25:**
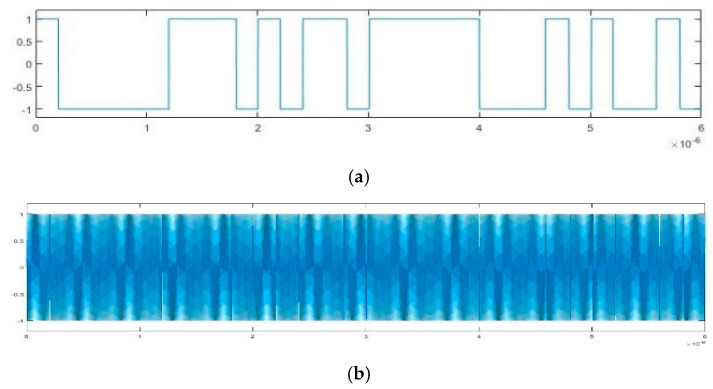
Estimated results based on Duffing oscillator array synchronization: (**a**) pseudo-random sequence; (**b**) reconstructed BPSK signal.

**Figure 26:**
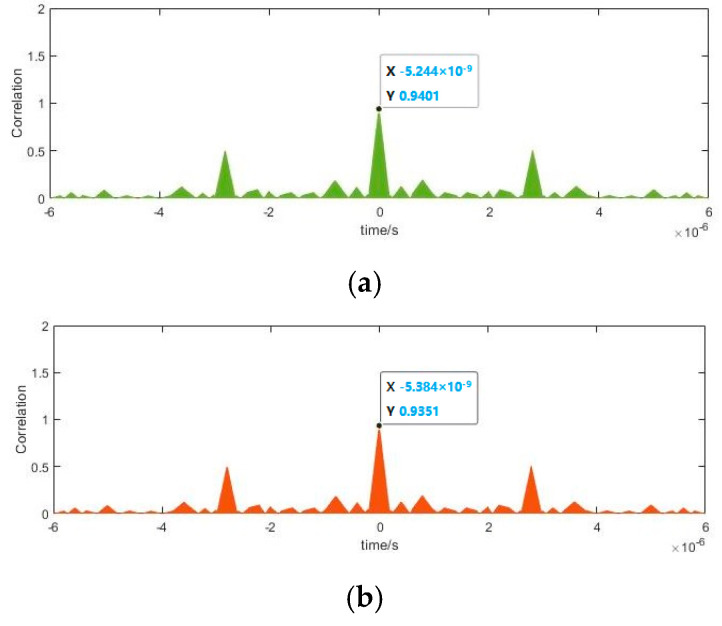
Correlated results: (**a**) implied periodicity; (**b**) pilot frequency array synchronization.

In order to realize the blind parameter estimation verification of BPSK signal based on Duffing oscillator, a semi-physical experiment system was established. A BPSK signal can be sent by the pseudo-random code phase modulation prototype and received by an antenna. After down-conversion, the signal with intermediate frequency is fed into the computer and estimated by Duffing oscillator system. Subsequently, the BPSK signal can be reconstruct according to the detected parameters. Finally, we test the effect of parameter estimation by correlative processing between the received intermediate frequency signal and the reconstructed one. Images of spectrum of emitted BPSK signal, time-domain of the signal after down-conversion, time-domain of the reconstructed signals based on Duffing oscillator methods and the experiment result of correlation processing are provided in Figure 22, Figure 23, Figure 24, Figure 25 and Figure 26. 

According to Figure 22, the frequency of emitted BPSK signal is approximately 10 GHz. We down-convert it to adjust the frequency to 100 MHz. The pseudo-random sequence of the emitted BPSK signal can be expressed as 100000111010110111110001010010 over a period of 6 × 10^–6^ s. As shown in Figure 23, each place where the phase flips represents a change in the pseudo-random sequence. After parameter estimation based on Duffing oscillator implied periodicity and pilot frequency array synchronization, the estimated carrier frequencies are 99.992 MHz and 99.9873 MHz, respectively. The estimated pseudo-random sequences and reconstructed BPSK signals obtained by these two Duffing oscillator methods are presented in Figure 24 and Figure 25. As shown in Figure 26, the maximum correlation coefficient based on two Duffing oscillator methods between the received signal and the reconstructed signal is 0.9401 and 0.9351, respectively, which proves the validity of these two methods.

## 5. Conclusions

In this letter, we have analyzed the output characteristics of BPSK signals through the Duffing oscillator and derived the multiplication relationship among the output of the Duffing oscillator, pseudo-random sequence, and cosine of the difference frequency, providing a theoretical basis for the blind estimation of BPSK signal. Based on it, we propose two blind estimation methods. Simulation and experiment results show that these two methods can estimate pseudo-random sequences and carrier frequencies with high accuracy and offer strong performance when SNR = –35 dB.

However, in our methods, pseudo-random sequences and carrier frequencies are closely related, if one parameter estimation has an error, it affects the other. Small errors have great effect on the estimation results. In addition, the binarization precision of the Duffing oscillator output signal in this paper is not high. In the future, we need to find a method to distinguish chaotic states in intermittent chaotic state of Duffing oscillator with less error, which can improve the overall estimation accuracy.

## Figures and Tables

**Table 1:** Relationship among the symbol of cos(Δ*ωt* + *φ*(*t*)), *φ*_i_, and *t*.

sgn(cos(Δ*ω* + *φ*(*t*)))	*φ*_i_ = 0	*φ*_i_ = *π*
*t*_1_ < *t* < *t*_2_	1	−1
*t*_2_ < *t* < *t*_1_ + 2*π*/|Δ*ω*|	−1	1

**Table 2:** Simulation parameters.

	Methods	Implied Periodicity	Pilot Frequency Array Synchronism
Parameters			
Duffing oscillator	*a*	1	1
	*b*	1	1
	*k*	0.5	0.5
	Amplitude	0.826	0.826
	Frequency	103 MHz, 98 MHz	97 MHz, 98 MHz, 101 MHz, 103 MHz
BPSK signal	Code Width	30 ns	30 ns
	Amplitude	0.6	0.6
	Frequency	100 MHz	100 MHz

**Table 3:** Simulation results of pseudo-random sequence correlation similarity coefficients at different SNR.

SNR/dB	Correlation Similarity Coefficients		
	Based on Implied Periodicity	Based on Array Synchronization	Based on Known Carrier Frequency
−10	0.9627	0.9725	0.9740
−20	0.9543	0.9703	0.9683
−30	0.9470	0.9627	0.9604
−35	0.9120	0.9156	0.9230
−40	0.8205	0.8208	0.8231

**Table 4:** Simulation results under different SNR.

SNR/dB	Carrier Frequency Estimation Relative Error (%)	
	Based on Implied Periodicity	Based on Array Synchronization
−10	0.0101	0.0142
−20	0.0112	0.0327
−30	0.0355	0.0491
−35	0.0828	0.0895
−40	0.2470	0.2794
